# Characterizing magnetically focused contamination electrons by off‐axis irradiation on an inline MRI‐Linac

**DOI:** 10.1002/acm2.13591

**Published:** 2022-03-25

**Authors:** Elizabeth Patterson, Bradley M. Oborn, Dean Cutajar, Urszula Jelen, Gary Liney, Anatoly B. Rosenfeld, Peter E. Metcalfe

**Affiliations:** ^1^ Centre for Medical Radiation Physics Wollongong NSW Australia; ^2^ Ingham Institute for Applied Medical Research Liverpool NSW Australia; ^3^ Illawarra Cancer Care Centre Wollongong Hospital Wollongong NSW Australia; ^4^ Illawarra Health Medical Research Institute University of Wollongong Wollongong NSW Australia

**Keywords:** dosimetry, electron contamination, inline MRI‐Linac, magnetic field, radiotherapy, skin dose

## Abstract

**Purpose:**

The aim of this study is to investigate off‐axis irradiation on the Australian MRI‐Linac using experiments and Monte Carlo simulations. Simulations are used to verify experimental measurements and to determine the minimum offset distance required to separate electron contamination from the photon field.

**Methods:**

Dosimetric measurements were performed using a microDiamond detector, Gafchromic^®^ EBT3 film, and MO*Skin* ^TM^. Three field sizes were investigated including 1.9 × 1.9, 5.8 × 5.8, and 9.7 × 9.6 cm^2^. Each field was offset a maximum distance, approximately 10 cm, from the central magnetic axis (isocenter). Percentage depth doses (PDDs) were collected at a source‐to‐surface distance (SSD) of 1.8 m for fields collimated centrally and off‐axis. PDD measurements were also acquired at isocenter for each off‐axis field to measure electron contamination. Monte Carlo simulations were used to verify experimental measurements, determine the minimum field offset distance, and demonstrate the use of a spoiler to absorb electron contamination.

**Results:**

Off‐axis irradiation separates the majority of electron contamination from an x‐ray beam and was found to significantly reduce in‐field surface dose. For the 1.9 × 1.9, 5.8 × 5.8, and 9.7 × 9.6 cm^2^ field, surface dose was reduced from 120.9% to 24.9%, 229.7% to 39.2%, and 355.3% to 47.3%, respectively. Monte Carlo simulations generally were within experimental error to MO*Skin* ^TM^ and microDiamond, and used to determine the minimum offset distance, 2.1 cm, from the field edge to isocenter. A water spoiler 2 cm thick was shown to reduce electron contamination dose to near zero.

**Conclusions:**

Experimental and simulation data were acquired for a range of field sizes to investigate off‐axis irradiation on an inline MRI‐Linac. The skin sparing effect was observed with off‐axis irradiation, a feature that cannot be achieved to the same extent with other methods, such as bolusing, for beams at isocenter.

## 1 INTRODUCTION

Image‐guided radiation therapy (IGRT) involves the use of imaging to delineate a target and normal structures within the treatment room, and to adapt treatment fields accordingly. Historically IGRT has involved megavoltage (MV) and kilovoltage (kV) x‐rays; however, limitations including poor soft tissue contrast and imaging dose have motivated the move toward magnetic resonance‐guided radiotherapy (MRgRT). MRgRT is possible with the integration of a magnetic resonance imaging (MRI) scanner and an x‐ray radiotherapy unit (Linac). These systems are known as MRI‐Linacs and offer superior soft tissue contrast that provide greater accuracy of tumor and organ delineation, superior motion tracking, and improved local tumor control; with an absence of imaging dose exposed to the patient.^1^

The development of an MRI‐Linac system introduces new and unique challenges relating to dose distribution changes that occur due to the presence of the magnetic field. Photon beams interact with matter via a multitude of interactions that liberate energized charged particles, such as electrons. These charged particles within a magnetic field undergo curved trajectories due to the Lorentz force. The magnitude of Lorentz forces on charged particles is dependent on magnetic field strength, particle energy, and the orientation of the incident beam relative to the magnetic field.^2^ For a transverse beam relative to a magnetic field, dosimetric effects include a decreased build up distance, asymmetric penumbra, electron streaming in air depositing dose outside the photon beam, and the electron return effect (ERE) that occurs at high‐to‐low density interfaces such as tissue‐lung and tissue‐air.^3^, ^4^, ^5^, ^6^, ^7^, ^8^, ^9^ Clinically, electron streaming and the ERE can be managed with the use of bolus and multiple treatment beam angles; however, it is possible that in‐patient air cavities, such as the trachea, can be affected.^8^, ^10^, ^11^, ^12^, ^13^, ^14^ With a magnetic field parallel or inline to the beam direction, these aforementioned dosimetric perturbations are reduced: no lateral dose shift, a reduction in penumbral width, and no ERE. However, a high field inline configuration does give rise to a high surface dose due to electron focusing along the central magnetic axis that can exceed the dose at d${}_{\max}$ leading to a loss of skin sparing.^15^, ^16^, ^17^ This becomes less pronounced for systems that have a lower magnetic field strength, such as the 0.5 T Aurora RT system (MagnetTx Oncology Solutions, Canada).^18^, ^19^

The interest in surface dose is motivated by skin reactions that arise immediately after radiotherapy and can continue for months and years.^20^ Radiation reactions, also classified as a radiation induced skin injury, can be severe and potentially require the expertise of wound care specialists, dermatologists, and plastic surgeons. Various investigations into excessive doses have been conducted using Monte Carlo simulations and experimental longitudinal MR‐IGRT systems. Based on these results, the need to mitigate severe skin reactions has become a principal concern. One approach to reduce high entrance dose was experimentally investigated and involved a 2‐cm‐thick acrylic beam spoiler/bolus placed 5 cm upstream from the surface of the phantom.^17^ This method was used to treat live rats that were treated for brain tumors within the Australian MRI‐Linac.^21^ Although this approach reduces surface dose, it also reintroduces electrons from the spoiler itself, leading to a higher surface dose well exceeding 100%, compared to normal x‐ray beam treatments that typically benefit from the skin sparing effect.^22^ The large surface dose from a high field inline MR‐linac would increase the likelihood of Grade 4 acute and late dermatological adverse reactions for the skin, which are considered the most severe and in some cases, have life‐threatening consequences.^23^, ^24^ Monte Carlo studies have explored the effectiveness of magnetic shielding to purge charged particles at the level of the multileaf collimator (MLC) and by placing a helium gas region between the linac and treatment surface to minimize the quantity of air‐generated contamination.^22^, ^25^

Surface dose can also be impacted by the radiofrequency (RF) receiving coil due to the close proximity relative to the anatomy of interest. Optimizing RF coil design minimizes the interactions with the incident beam that can produce secondary electrons and consequently contribute to an increased surface dose.^26^, ^27^, ^28^

An earlier investigation by our group considered irradiating off‐axis on a high field 1.5 T inline 6 MV MRI‐Linac and found that contamination electrons favor deposition along the magnetic isocenter of the MRI bore.^29^ The benefits of off‐axis irradiation for an inline MRI‐Linac include the return of the skin sparing effect and a reduction of high surface doses at the treatment site. A disadvantage is that by irradiating a treatment site off‐axis away from the isocenter of the MRI, image quality can suffer. In particular, gradient non‐linearity (GNL) is known to increase with distance from isocenter and cause geometric distortions at extreme margins of MR images.^30^ For off‐axis treatment, the beam should be offset by the smallest distance from isocenter while still achieving electron contamination separation. This would reduce GNL effects that are maximized with increasing distance from isocenter that can impact tumor localization and the radiotherapy treatment. It is expected that the TPS would be able to accurately model off‐axis treatment in a similar way when a tumor cannot be aligned to the machine isocenter. This paper further investigates this novel concept of off‐axis irradiation with point dose detector measurements and Monte Carlo simulations that will provide further clarification on the suitability of this method in the pursuit for a clinically useful beam.

The presence of a magnetic field impacts electron paths within detectors, and the readout can vary compared to an identical setup in the absence of a magnetic field. The composition of the detector, relative orientation within the magnetic field, field strength, beam energy, and field size can impact the response of a detector, potentially requiring a magnetic field correction factor, and particularly so for ionization chambers.^31^, ^32^, ^33^, ^34^ The response of several detectors has been investigated including ionization chambers (single and array^35^), film,^36^, ^37^, ^38^ thermoluminescent dosimeters (TLD),^38^ novel plastic scintillation detectors,^39^ a 4D gel dosimeter,^40^ and solid‐state detectors such as the diamond and diode detectors.^41^ The effects of dose perturbations are reduced in longitudinally orientated MRI‐Linacs, which has also been observed with detectors such as the PTW 60003 diamond detector where little to no dose difference occurs for field strengths up to 1.5 T.^41^ The air‐filled cavity of ionization chambers lend them to be more susceptible to magnetic field dose influences; however, for longitudinal fields, the change with respect to zero field is minimal.^33^

## 2 MATERIALS AND METHODS

The Australian MRI‐Linac includes a split bore 1 T MRI scanner (Agilent, UK) and a 6 MV linear accelerator Linatron‐MP (Varex, USA) coupled with a stand‐alone Millennium MLC (Varian, USA). Unique to this system, the Linatron and MLC are mounted on rails such that the source‐to‐surface distance (SSD) can be varied between 1.8 and 3.2 m. This allows the Linatron components (i.e., electron gun, waveguide, target, MLC) to be moved into regions of various magnetic field strengths. In this study, an SSD of 1.8 m was used, which is one of the two SSD positions the system has been commissioned. At this SSD position, the linac is within the fringe field of the magnet that impacts electron transport and consequently beam generation resulting in a higher generation of secondary electron contamination. The shorter SSD of 1.8 m was chosen for this study as the relative surface dose compared to the extended SSD is larger and acts as a “worst case” scenario of electron contamination.^17^

Experimental depth dose measurements were acquired with EBT3 Gafchromic film (Ashland Inc, USA), a PTW microDiamond type 60019 detector, and MO*Skin* ^TM^. Gafchromic EBT3 film was included in this study due to its high spatial resolution, making it useful for two‐dimensional dose distribution analysis. Although some studies have investigated EBT3 Gafchromic film dosimetry exposed to a magnetic field with varying conclusions and inconsistent results, the general consensus is that EBT3 film is a suitable dosimeter for MR‐linac use where insignificant changes in dose occur compared to 0 T irradiation.^38^, ^42^, ^43^

The effective point of measurement (EPOM) of EBT3 film is defined as the middle of the sensitive layer, which is 0.139 mm from the surface of the film. A Dose1 electrometer (Scanditronix Wellhofer, Germany) was connected for microDiamond read‐out. The EPOM for the microDiamond is 1 mm below the surface as specified by the vendor^44^ and was irradiated face‐on. The MO*Skin* ^TM^ is a metal oxide semiconductor field effect transistor (MOSFET) detector developed by the Centre of Medical Radiation Physics (CMRP) with the capability of fast and reproducible surface dose measurements at a water equivalent depth (WED) of 70 μm. The small physical size and volume of the MO*Skin* ^TM^ makes it a suitable choice for high dose gradient measurements, such as those that occur in a inline MRI‐Linac.^16^, ^22^ The MO*Skin* ^TM^ has been designed to directly compare to the ICRP recommendation of the most radiosensitive layer of human skin located at a depth of 0.07 mm beneath the surface of the skin.^45^, ^46^

For simulations, the Monte Carlo simulation toolkit, Geant4 version 10.06.01, was used. The charged particle step limit was set to 0.1 mm for accurate magnetic field transport. Existing phase space files located at a plane above the MLCs were used as the particle gun for *B* = 1 T and *B* = 0 T simulations. Scoring voxels were set to 1 mm spanning a 30 × 30 × 30 cm^3^ water phantom at an SSD of 1.8 m. MLC files were adjusted to position the field sizes far off‐axis (10 cm), minimally off‐axis (2.1 cm), and at isocenter. Adjoining MLC leaf pairs for all simulations met at the central axis of the MLC bank. The MLC leaf leakage can be seen in Figure 3 and labeled using an overlayed, vertical black line. Monte Carlo simulations were used to verify experimental measurements and also determine, via trial and error, the minimum offset distance for each of three field sizes to separate electron contamination. A method to absorb the separated electron contamination was also investigated and involved the use of a 2‐cm‐thick water spoiler placed at the border edge of each field size, closest to the central magnetic axis. For nonoffset central fields, the typical definition of $d_{\max}$ normalization becomes redundant due to an absence of photon build‐up caused by electron contamination. Central field simulations with *B* = 0 T were used to determine $d_{\max}$ and from this the dose at $d_{10\text{cm}}$, which is not impacted by electron contamination, was used to normalize *B* = 1 T Monte Carlo data.

**FIGURE 1:**
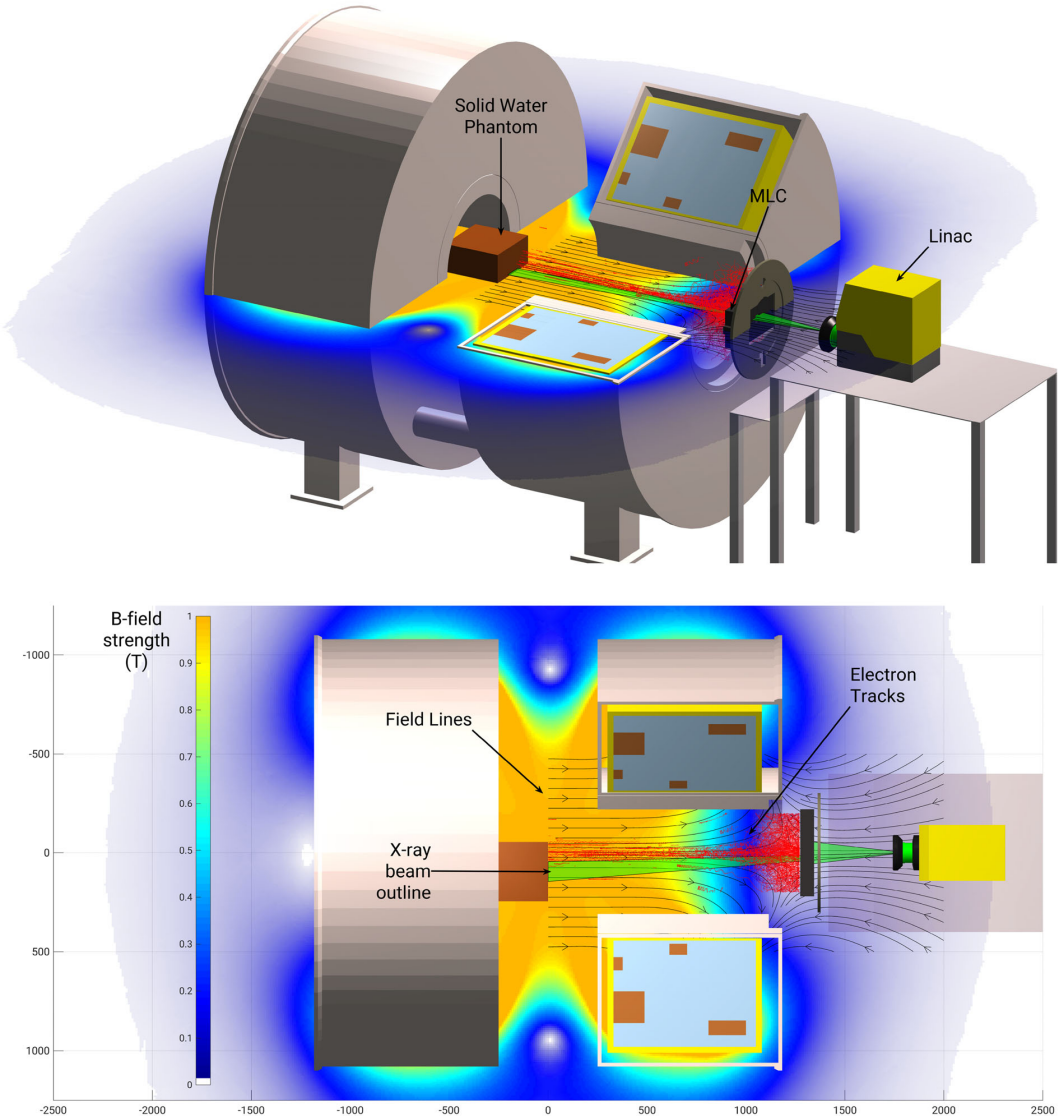
Schematic of the Australian MRI‐Linac system. Top: Side view showing a split‐bore MRI, Linac, MLC, and solid water phantom. Bottom: Top view of the offset phantom where the overlay grid indicates the dimensions of the system (mm). A color map ranging 0–1 T has been included to show the relative intensity of the magnetic field. The x‐ray beam and electron tracks are represented by a green outline and red tracks, respectively

**FIGURE 2:**
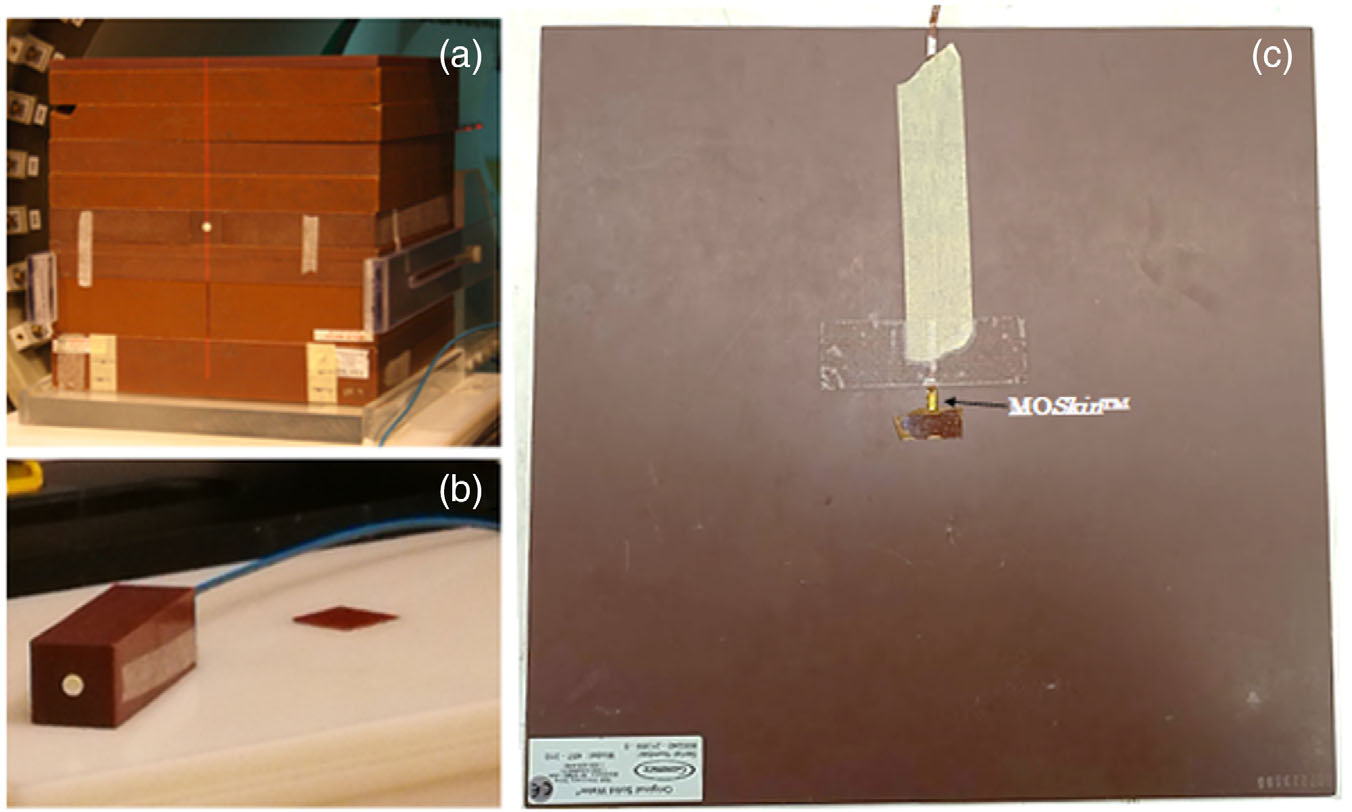
Experimental setup: (a) Solid water phantom with microDiamond detector at surface, (b) microDiamond phantom, and (c) MO*Skin* ^TM^ solid water phantom

**FIGURE 3:**
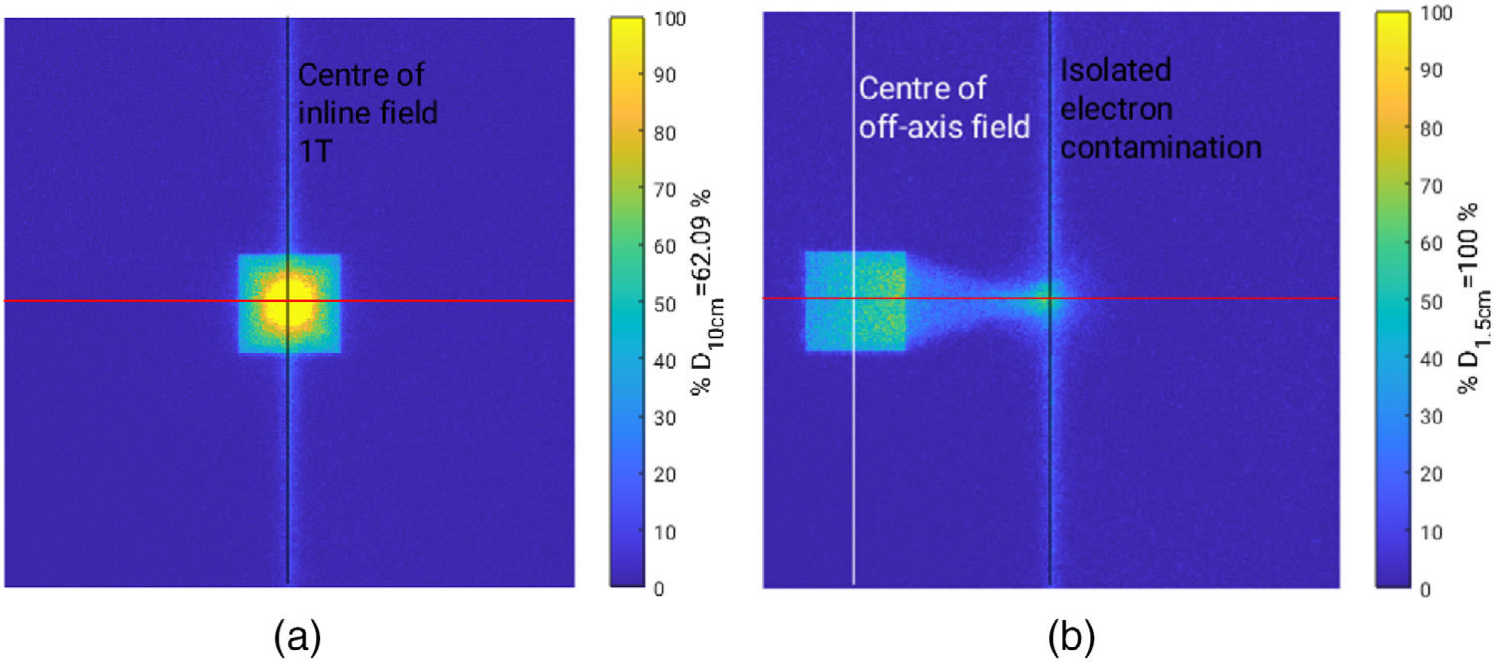
Labeled Monte Carlo 2D dose maps for field size 5.8 × 5.8 cm^2^ at the surface to indicate locations of PDD measurements. (a) 2D dose map at the surface of the water phantom that indicates the central axis for a central beam (black vertical line), denoted as “1 T” for PDD plots. The horizontal red line indicates the position of cross plane profiles. (b) 2D dose map at the surface of the water phantom that indicates the center of an off‐axis field (white vertical line) and magnetic isocenter axis that coincides to the leakage of adjoining MLC leaf pairs (black vertical line)

### 2.1 Experimental setup

For all measurements, a 30 × 30 × 30 cm^3^ solid water block phantom was positioned at an SSD of 1.8 m as shown in Figure 1 using the rooms lasers. For each field size investigated, a central and off‐axis MLC file was used. The central MLC file produced a field located at the central axis, with an example of this shown in Figure 3a. The off‐axis file produced a field 9.5 cm from the central axis, similar to what can be seen in Figure 3b where a 10 cm distance was used. For the microDiamond and MO*Skin* ^TM^, a solid water piece was manufactured to minimize air gaps. For the MO*Skin* ^TM^, a 1 mm recess was cut into a solid water block. Figure 2 includes the microDiamond‐specific phantom that was manufactured to fit the detectors width and length, and irradiated perpendicular to the length of the detector as per the manufacturer recommendations.^44^ PDD measurements were performed for a range of field sizes, including 1.9 × 1.9, 5.8 × 5.8, and 9.7 × 9.6 cm^2^ defined at SSD = 1.8 m. At an extended SSD of 1.8 m, the MLC‐defined fields are subject to a magnification factor along with scaling of the leaf opening shape that can result in nonstandard square field sizes at isocenter. Film was placed at selected depths within the solid water phantom that included 1, 15, 20, and 50 mm, whereas the MO*Skin* ^TM^ and microDiamond were used to obtain high resolution, near‐surface measurements with the use of 50 μm polyimide (kapton tape), added layer by layer above the MO*Skin* ^TM^, and depths to 180 mm using various solid water phantom thicknesses. Care was taken to minimize air gaps around the film as this is known to cause minor dose errors.^47^

EBT3 film was calibrated using a 6 MV photon beam following the recommendations in the AAPM Task group 55 report.^48^ Films were scanned using an EPSON Perfection V800 flatbed scanner (Epson, Japan), with a resolution of 72 dpi and 48 bit RGB color depth. The red color channel was used to analyze the films' optical density, and image registration was performed using ImageJ. Film uncertainties were calculated according to recommendations of Marroquin.^49^ Film profiles were normalized to the dose at the center of the nonflat beam for film located at 1.5 cm depth within the phantom. Three readings were obtained and averaged for each microDiamond and MO*Skin* ^TM^ measurement. Quoted uncertainties of MO*Skin* ^TM^ and microDiamond measurements are the sum of statistical error, calculated as 95% confidence interval normalized to d${}_{\max}$, and systematic errors, represented as 3% to estimate the effect of field intensity variation and detector positioning. Systematic errors relating to field intensity variations only apply to point dose measurements and are therefore not included when quoting film uncertainties. For central field measurements where electron contamination impacts typical photon build up, d${}_{\max}$ normalization becomes redundant because the maximum dose is found at the surface. To compare central and off‐axis fields, MO*Skin* ^TM^ measurements were normalized relative to the dose of the off‐axis field at a depth of 5 cm. At this depth, electron contamination has been attenuated and the PDD's curves of the central and off‐axis fields align.

## 3 RESULTS

**FIGURE 4:**
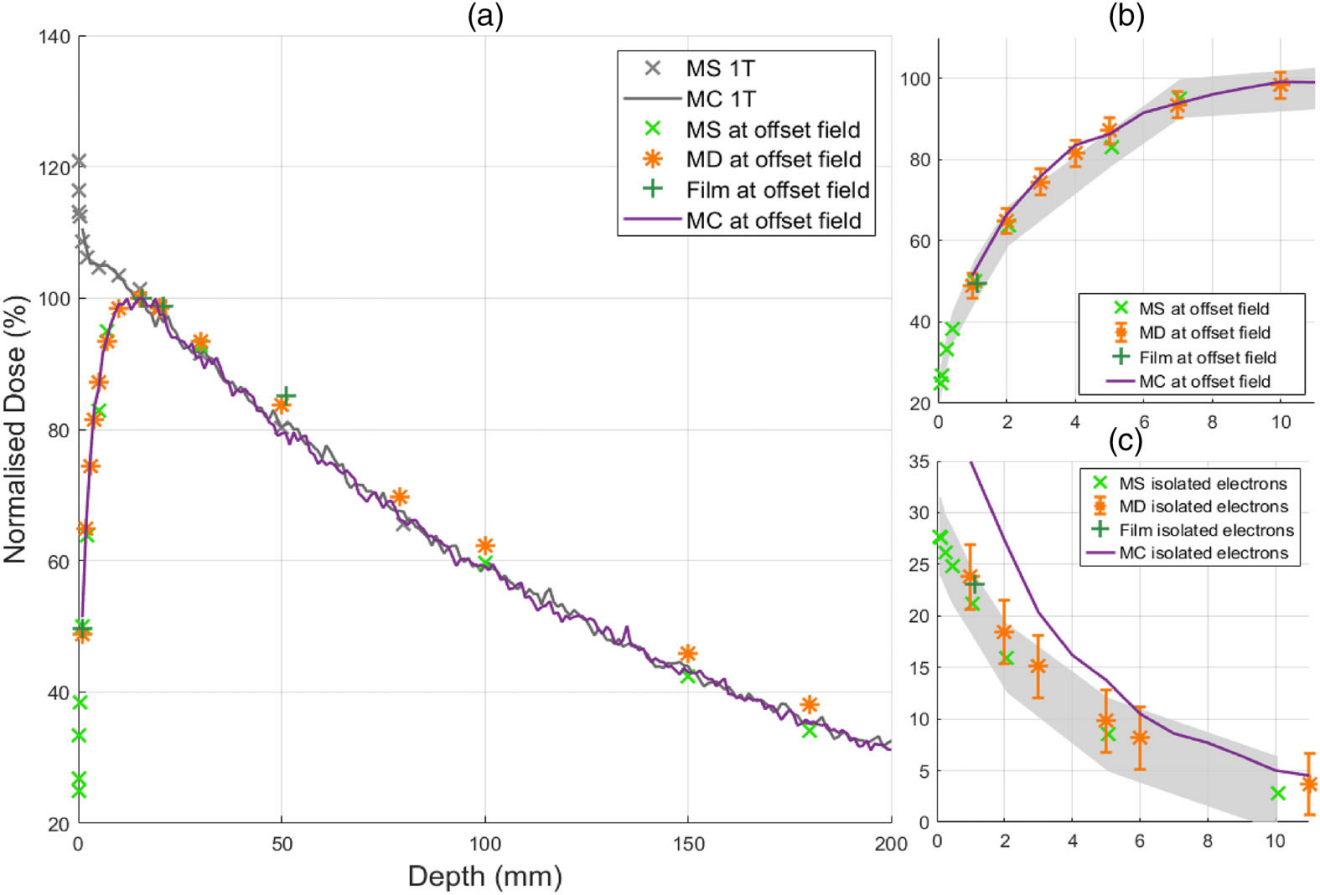
Depth dose curves for 1.9× 1.9 cm^2^ field measured with MO*Skin* ^TM^(× ), microDiamond (*), EBT3 film (+), and Monte Carlo simulations (–). (a) PDD of the off‐axis field and centered field, denoted as 1 T within the figure legend, with a surface dose of 24.9% and 120.9%, respectively. (b) First 10 mm PDD for off‐axis field measurements. (c) First 10 mm PDD at the central magnetic axis during off‐axis irradiation with a maximum surface dose of 27.7%. Shaded gray error bars in (b) and (c) represent MO*Skin* ^TM^ uncertainties and conventional error bars for microDiamond. For field size 1.9× 1.9 cm^2^, maximum uncertainty of MO*Skin* ^TM^, microDiamond, and film was 7.0%, 3.2%, and 4.0%

A summary of the surface dose as measured by the MO*Skin* ^TM^ is shown in Table 1. Figure 3 depicts in‐plane profiles to aid in understanding the location of various depth dose measurements including “1 T,” which corresponds to the vertical black line in Figure 3a. In Figure 3b, “offset field” and “isolated electrons” correspond to the vertical white and black lines. The rate at which the surface dose increases with field size is significant for depth dose measurements at isocenter, denoted as “centered field surface dose, 1 T” in Table 1. PDD results for the smallest field size, 1.9× 1.9 cm^2^, at all three locations within the MRI bore are shown in Figure 4. The reduction of air‐generated contamination electrons from the off‐axis field produces a typical MV photon build‐up curve, whereas ordinarily for a high field inline MRI‐Linac, a steep dose gradient occurs in the first few centimeters. With an increase in field size, electron focusing along the central magnetic axis where electron contamination can be found creates a large surface dose exceeding 350% as shown in Figure 6.

**TABLE 1:** Surface dose for centered fields and fields off‐axis by 9.5 cm from isocenter, measured with the MO*Skin* ^TM^ detector

Field size	Centered field surface dose, 1 T	Off‐axis field surface dose
(cm^2^)	(% $d_{5\text{cm}})$	(% $d_{\max})$
1.9× 1.9	120.9	24.9
5.8× 5.8	229.7	39.2
9.7× 9.6	355.3	47.3

**FIGURE 5:**
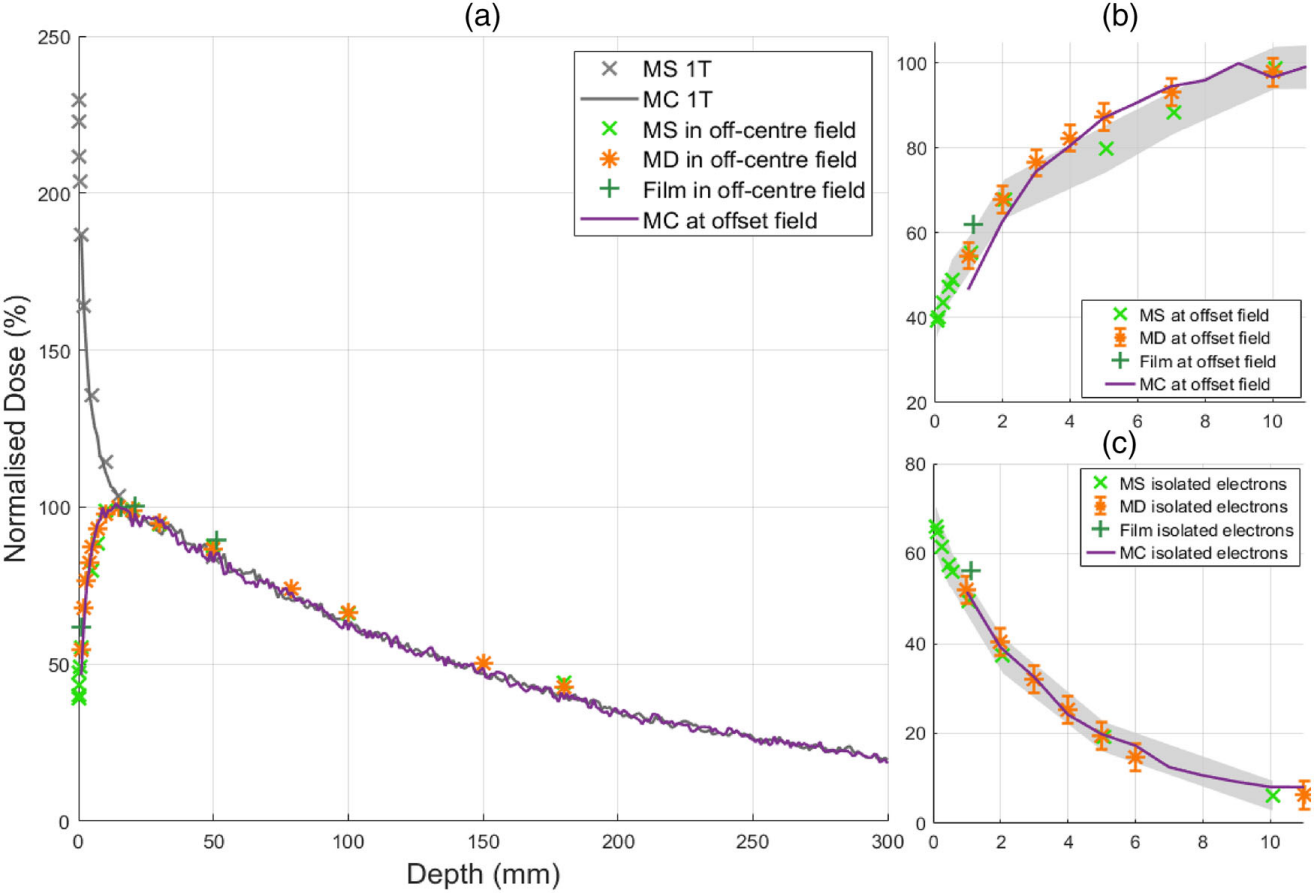
Depth dose curves for 5.8× 5.8 cm^2^ field measured with MO*Skin* ^TM^(× ), microDiamond (*), EBT3 film (+), and Monte Carlo simulations (–). (a) PDD of the off‐axis field and centered field, denoted as 1 T within the figure legend, with a surface dose of 39.2% and 229.7%, respectively. (b) First 10 mm PDD for off‐axis field measurements. (c) First 10 mm PDD at the central magnetic axis during off‐axis irradiation with a maximum surface dose of 66.1%. Shaded gray error bars in (b) and (c) represent MO*Skin* ^TM^ uncertainty and conventional error bars for microDiamond. For field size 5.8× 5.8 cm^2^, maximum uncertainty of MO*Skin* ^TM^, microDiamond, and film was 6.0%, 3.3%, and 4.0%

**FIGURE 6:**
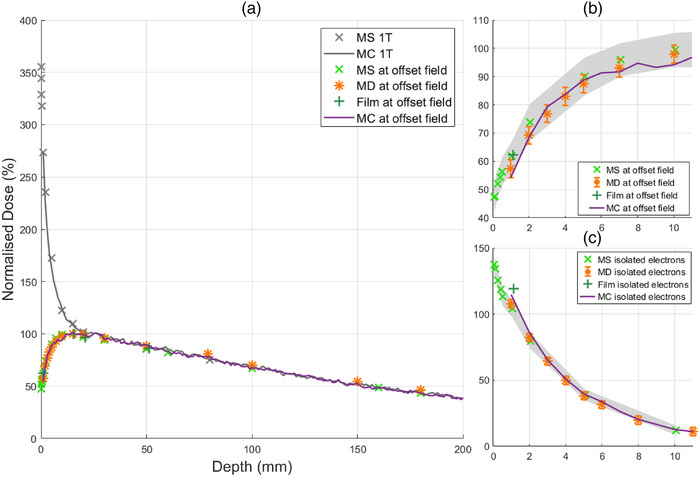
Depth dose curves for 9.7× 9.6 cm^2^ field measured with MO*Skin* ^TM^(× ), microDiamond (*), EBT3 film (+), and Monte Carlo simulations (–). (a) PDD of the off‐axis field and centered field, denoted as 1 T within the figure legend, with a surface dose of 47.3% and 355.3%, respectively. (b) First 10 mm PDD for off‐axis field measurements. (c) First 10 mm PDD at the central magnetic axis during off‐axis irradiation with a maximum surface dose of 137.2%. Shaded gray error bars in (b) and (c) represent MO*Skin* ^TM^ uncertainties and conventional error bars for microDiamond. For field size 9.7× 9.6 cm^2^, maximum uncertainty of MO*Skin* ^TM^, microDiamond, and film was 7.2%, 3.7%, and 4.0%

**FIGURE 7:**
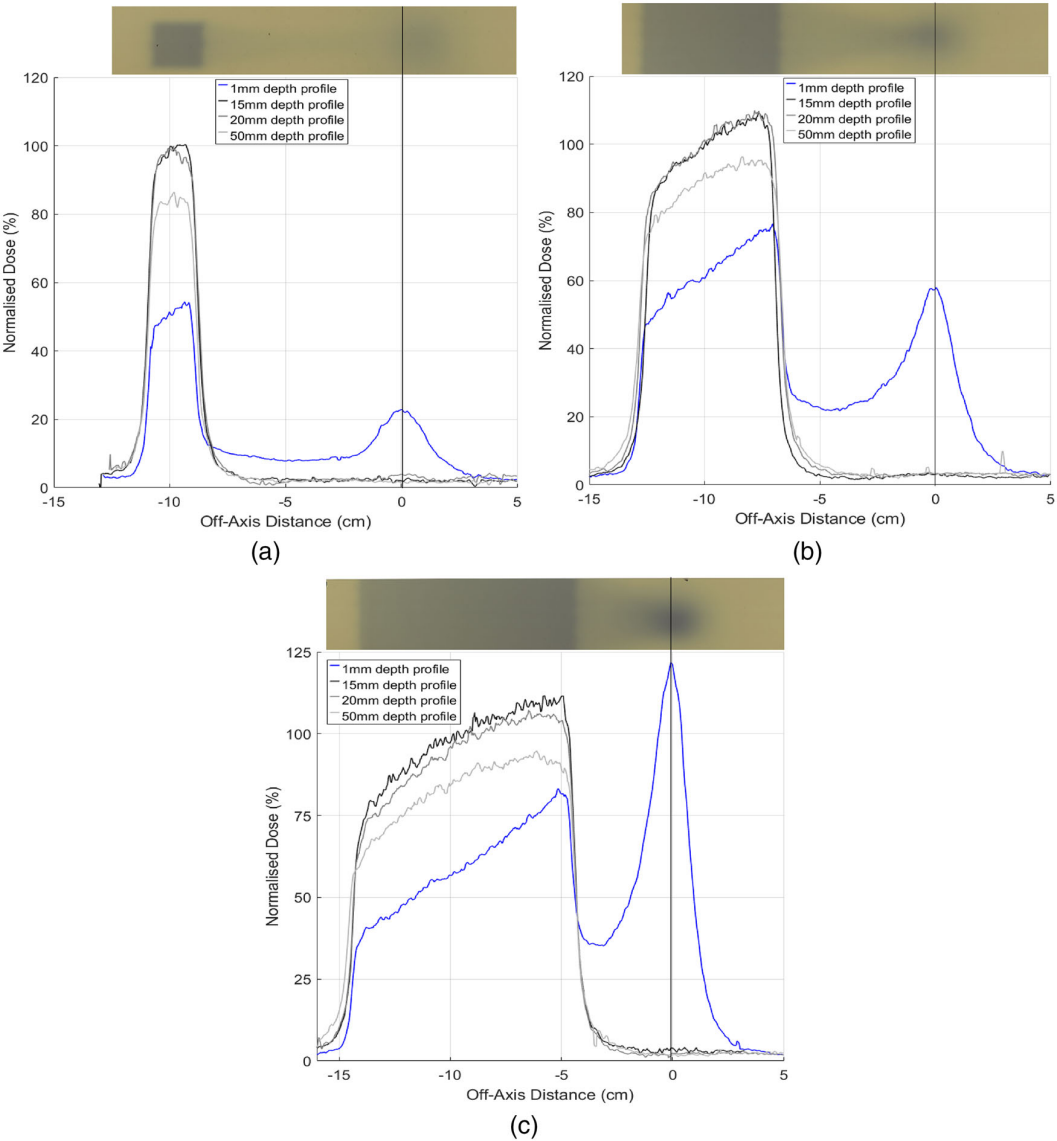
Film profiles at depths 1, 15, 20, and 50 mm for field sizes (a) 1.9× 1.9 cm^2^, (b) 5.8× 5.8 cm^2^, and (c) 9.7× 9.6 cm^2^. Above each profile graph is a raw film scan prior to image processing. All film data were normalized at the center of the off‐axis field at a depth of 1.5 cm. The vertical black line overlaid on each plot indicates the central magnetic axis (isocenter)

To determine the magnitude and size of the electron contamination along the central magnetic axis that occurs with off‐axis irradiation, film profiles were taken at 1, 15, 20, and 50 mm depths for all three field sizes and normalized to the center of the off‐axis field at 15 mm. Film profiles for each field size investigated are shown in Figure 7. The beam profile is nonflat and asymmetric for each field size, with a leading edge closest to the magnetic isocenter where electrons deposit energy. Profiles for the 1.9× 1.9 and 5.8× 5.8 cm^2^ fields show that dose from contamination electrons at magnetic isocenter is lower than in field dose. As the field size increases, electron dose at the surface begins to surpass photon surface dose as shown for the largest field size, 9.7× 9.6 cm^2^, in Figure 7c.

**FIGURE 8:**
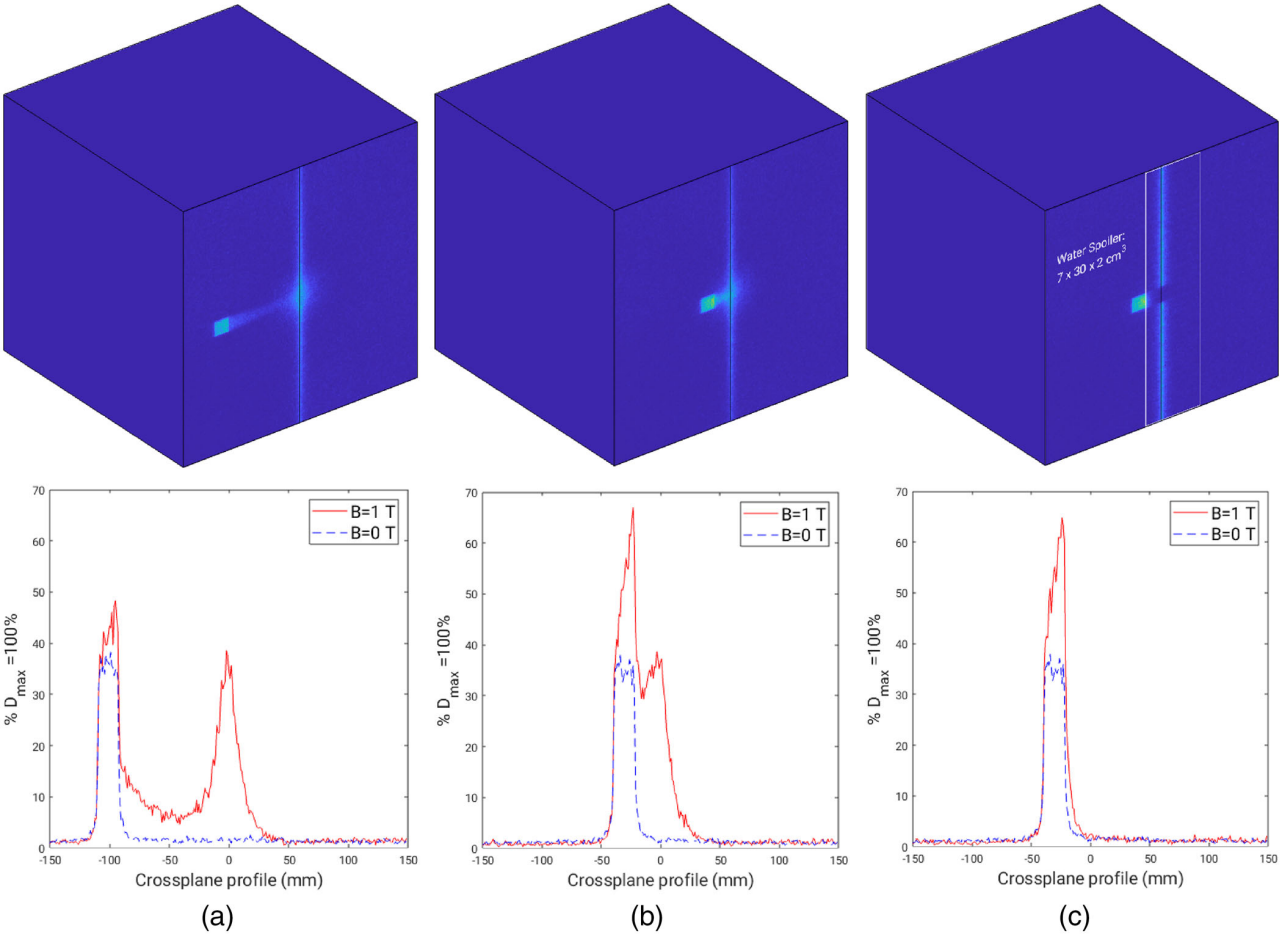
Monte Carlo simulations for a 1.9× 1.9 cm^2^ field. (a) *B* = 1 T, 2D dose map and crossplane profiles for 10 cm off‐axis field at 1 mm depth for *B* = 1 T and *B* = 0 T. (b) *B* = 1 T, 2D dose map and crossplane profiles for a minimum off‐axis distance of 3.1 cm from the center of the field to the central axis at 1 mm depth for *B* = 1 T and *B* = 0 T. (c) *B* = 1 T, 2D dose map and crossplane profiles at 1 mm depth for a minimum offset distance of 3.1 cm from the center of the field to the central axis with the addition of a water spoiler, outlined in white, for *B* = 1 T and *B* = 0 T

**FIGURE 9:**
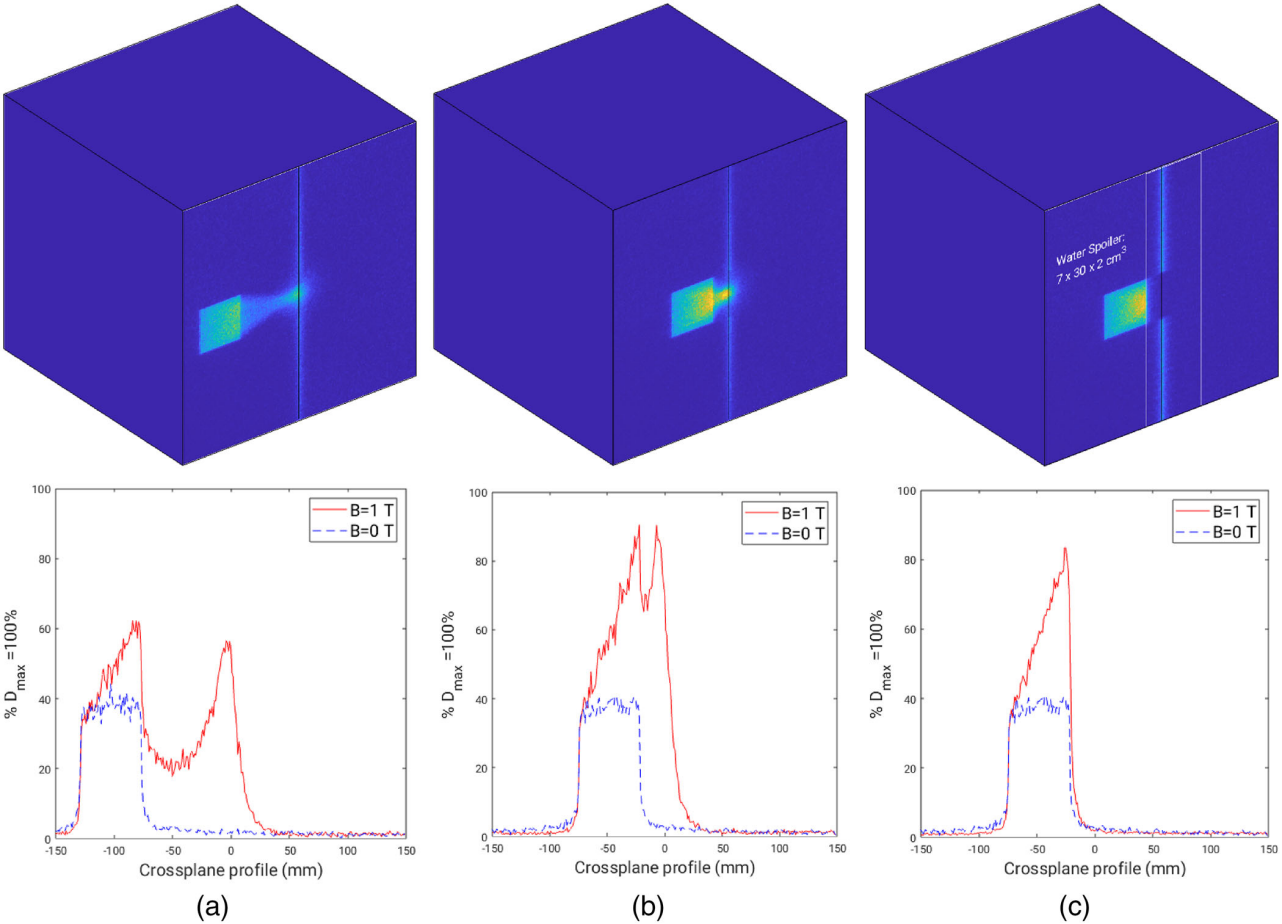
Monte Carlo simulations for a 5.8× 5.8 cm^2^ field. (a) *B* = 1 T, 2D dose map and crossplane profiles for 10 cm off‐axis field at 1 mm depth for *B* = 1 T and *B* = 0 T . (b) *B* = 1 T, 2D dose map and crossplane profiles for a minimum off‐axis distance of 4.8 cm from the center of the field to the central axis at 1 mm depth for *B* = 1 T and *B* = 0 T. (c) *B* = 1 T, 2D dose map and crossplane profile at 1 mm depth for a minimum offset distance of 4.8 cm from the center of the field to the central axis with the addition of a water spoiler, outlined in white, for *B* = 1 T and *B* = 0 T

**FIGURE 10:**
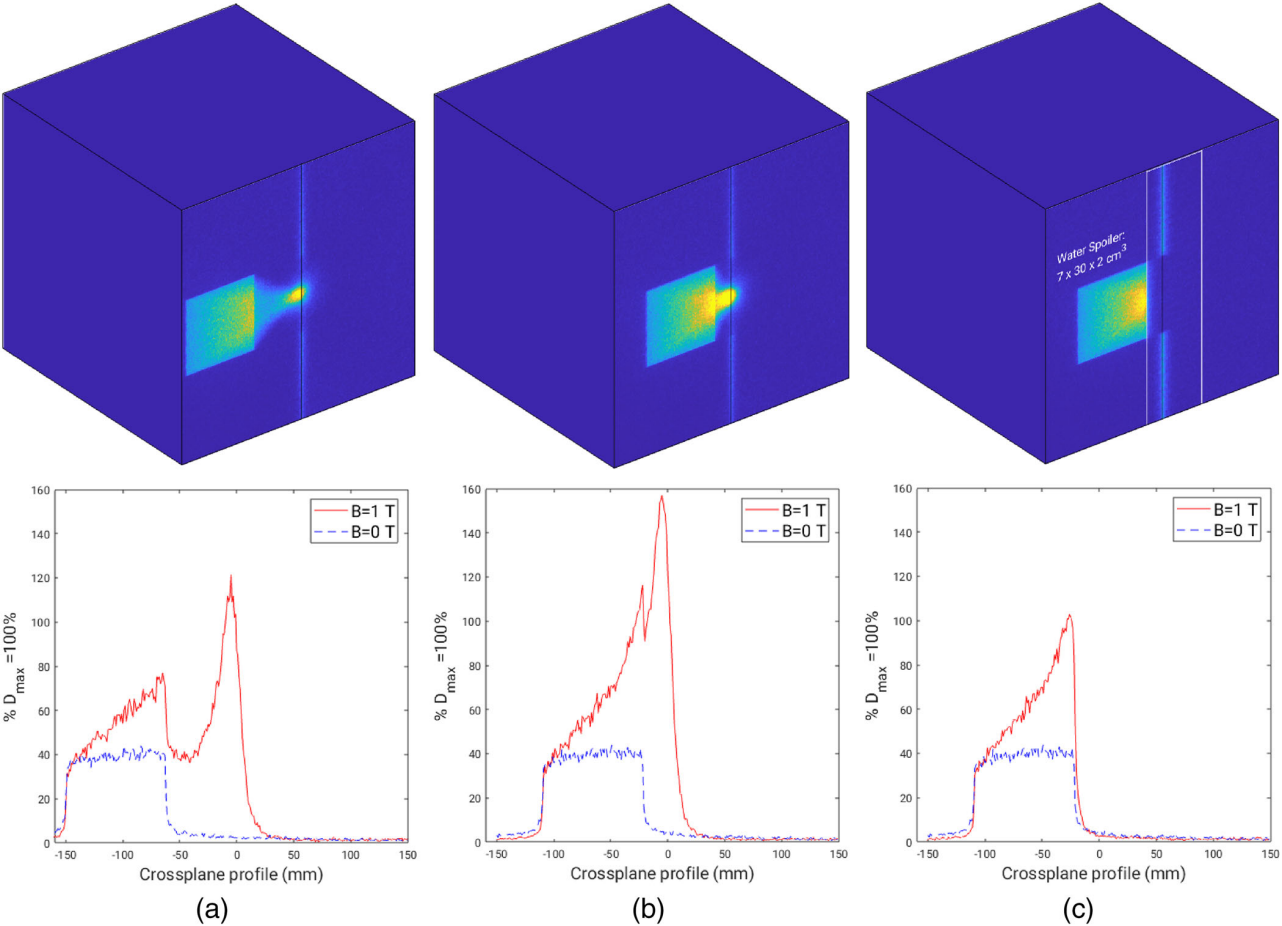
Monte Carlo simulations for a 9.7× 9.6 cm^2^ field. (a) *B* = 1 T, 2D dose map for *B* = 1 T and crossplane profiles for the 10 cm off‐axis field at 1 mm depth for *B* = 1 T and *B* = 0 T. (b) *B* = 1 T, 2D dose map and crossplane profiles for a minimum off‐axis distance of 6.5 cm from the center of the field to the central axis at 1 mm depth for *B* = 1 T and *B* = 0 T. (c) *B* = 1 T, 2D dose map and crossplane profiles at 1 mm depth for a minimum offset distance of 6.5 cm from the center of the field to the central axis with the addition of a water spoiler, outlined in white, for *B* = 1 T and *B* = 0 T

Monte Carlo 2D dose maps and profiles are shown in Figures 8, 9, 10. Figure 8 includes simulations for a 1.9× 1.9 cm^2^ field offset by 10 and 3.1 cm, relative to the center of the field and the central magnetic axis. The minimum offset distance between the center of the off‐axis field and the central magnetic axis was found to be 3.1 cm. This offset corresponds to a distance of 2.1 cm that separates the field edge to the central magnetic axis. The 2.1 cm offset distance will be quoted when referring to the minimum offset found for each field size as opposed to the distance between the center of field and central magnetic axis. Crossplane profiles at the first dose voxel layer, corresponding to a relative depth of 1 mm, can be seen in Figures 8a–c. Simulations with the magnetic field on, *B* = 1 T, and magnetic field off, *B* = 0 T, demonstrate an asymmetry of the off‐axis field that only appears when the magnetic field is present. This indicates that a gradient of electron contamination exists across the entire phantom. This is particularly prominent when the beam is minimally offset as shown in Figure 8b. A water spoiler, outlined in Figure 8c, demonstrates how electrons outside the field can be attenuated and lead to a near zero entry dose closely surrounding the central magnetic axis. Similar trends were seen in profiles produced for the larger field sizes, 5.8× 5.8 and 9.7× 9.6 cm^2^, as shown in Figures 9 and 10; however, the field asymmetry gradient is steeper due to the greater production of contamination electrons. This means that at surface and near surface depths, an off‐axis field is nonflat whereby the dose at each field edge (crossplane direction) can vary up to 60% as shown in Figure 10c.

## 4 DISCUSSION

The Australian MRI‐Linac system creates an environment where the fringe field focuses contamination electrons along the central magnetic axis, resulting in excessive entrance dose that has the potential to cause skin toxicity. To mitigate the impact of electron focusing on an inline MRI‐Linac, off‐axis irradiation was explored. By off‐setting the primary beam from the central magnetic axis, contamination electrons acted on by the fringe field are separated. Electron focusing, and consequently diameter of the electron hot spot, is proportional to field size used as larger fields generate a higher quantity of electron contamination. With off‐axis irradiation, large and open field sizes become limited because the separation between the edge of the electron hot spot and field exceeds the physical dimensions of the MRI bore. For example, a beam 11.8× 11.5 cm^2^ creates an electron hot spot diameter of 8 cm.^50^ Considering this, the maximum field size used for this study was 9.7× 9.6 cm^2^ and offset by 9.5–10 cm from the central magnetic axis (isocenter). For consistency, a constant field offset distance was used for all experimental measurements. A slightly larger and simplified offset of 10 cm was used for Monte Carlo simulations that would have little impact on the PDD comparisons to experimental measurements, when considering the systematic error of detector positioning.

For conventional measurements inline at the central axis, MO*Skin* ^TM^ surface doses for fields 1.9× 1.9, 5.8× 5.8 , and 9.7× 9.6 cm^2^ were found to be 120.9%, 229.7%, and 355.3%. With an off‐axis field, surface doses were reduced to 24.9%, 39.2%, and 47.3% respectively. Monte Carlo simulations agreed within experimental error to measurements as shown in Figure 5 with the exception of isolated electron measurements for the smallest field size, as shown in Figure 4c. This is likely a result of the low dose rate of contamination electrons originating from a smaller field size, and systematic error of detector positioning. MO*Skin* ^TM^, microDiamond, and film measurements at all field sizes showed a rapid decrease of contamination electron dose within solid water, particularly at 2 cm depth. Monte Carlo simulations were used to determine the minimum off‐axis distance to separate electron contamination from the primary photon beam along with dimensions and placement of a water spoiler to absorb electron contamination. The minimum offset determined with Monte Carlo simulations was found to be 2.1 cm. Figures 8, 9, 10 include a 2D dose map and crossplane profiles of the first dose voxel (equivalent to a depth of 1 mm) within the 30×30×30 cm^3^ water phantom for *B* = 1 T and *B* = 0 T scenarios. For each field size, a maximum field offset of 10 cm, a minimum offset, and minimum offset with a water spoiler, were simulated. The optimum spoiler dimension to reduce electron contamination dose to near zero was found to be 7×30×2 cm^3^ that corresponds to a width of 7 cm, height of 30 cm, and thickness of 2 cm, as shown in Figures 8, 9, and 10c with a white outline. The spoiler was positioned according to the in‐plane edge of the spoiler and aligned to the edge of each field when offset 2.1 cm from the central magnetic axis. Additionally, the spoiler was positioned +2 cm from the surface of the 30 × 30 × 30 cm^3^ with no air gaps.

Although a majority of electrons can be removed with the use of a water spoiler around the central magnetic axis, a gradient across each field exists for the *B* = 1 T case only, as shown in Figures 8, 9, and 10c. This is likely caused by the production of secondary electrons in air just above the phantom and photon interactions within the phantom. While the spoiler is able to remove secondary electrons originating well above the phantom surface that deposit dose around the central magnetic axis, electrons produced close in proximity to the phantom still exist. The asymmetry of the off‐axis field becomes less pronounced with depth as was seen with film profiles in Figure 7 where the fields flatten with depth. Despite the unavoidable asymmetry of the off‐axis field due to localized electron contamination, the entry dose is still significantly lower compared to when the field is centered at the central axis, as evident in Table 1. For clinical implementation, an FFF compatible TPS would be able to determine regions of the body at risk of dose from contamination electrons. A water spoiler could be incorporated into the TPS and placed at these regions that surround the treating beam. Note if the spoiler was placed in‐field, it would act as a bolus for the treatment that may be beneficial for superficial tumors. The Australian MR‐Linac has primarily been used for fixed gantry irradiation; however, human rotation is actively being investigated for future human trials.^51^ Off‐axis treatment could be a suitable treatment option to avoid excessive skin dose and is unlikely to require lengthened TPS computation time or exhibit image distortion for all fields, excluding those that are large or open.

## 5 CONCLUSION

A high‐resolution MO*Skin* ^TM^ detector, microDiamond, and film were used to measure PDDs and 2D dose maps for off‐axis irradiation at the Australian MRI‐Linac. Monte Carlo simulations were used to verify experimental measurements and determine the minimum field offset distance to separate central axis electron contamination from the primary beam. Monte Carlo simulations demonstrated that secondary electrons that came into contact with a 2‐cm‐thick water spoiler were fully attenuated prior to the phantom surface; however, the gradient electron contamination that does not interact with the spoiler will result in a nonflat field. With depth, the field will flatten as the electrons become attenuated.

A significant reduction of dose, as great as −308% for a 9.7× 9.6 cm^2^ field, at the beam entry site can be achieved with off‐axis irradiation and could be considered as an alternative method for high field, inline MR‐Linac patient treatments. Incorporating a water spoiler would shield the patient's skin from unnecessary electron contamination dose that could cause skin toxicity away from the treatment beam. For each field size, spoiler dimensions should be considered to ensure that the spoiler does not cover the treatment area; otherwise, it would act as a bolus and increase skin dose.

## CONFLICT OF INTEREST

The authors have declared no conflict of interest.

## AUTHOR CONTRIBUTION STATEMENT

E. Patterson, D. Cutajar, and P. Metcalfe designed the study. E. Patterson performed the experiments and wrote the manuscript. B. Oborn generated a key figure for the manuscript, assisted in Monte Carlo analysis, and assisted in writing the revised manuscript. D. Cutajar and U. Jelen partly assisted in the acquisition of experimental measurements. G. Liney conceived the method in an earlier publication. A. Rosenfeld provided advice on the MO*Skin* ^TM^ dosimeter response characteristics. Authors E. Patterson, G. Liney, B. Oborn, A. Rosenfeld, and P. Metcalfe contributed to the final manuscript.
